# Prediction of NOx Emissions in Thermal Power Plants Using a Dynamic Soft Sensor Based on Random Forest and Just-in-Time Learning Methods

**DOI:** 10.3390/s24144442

**Published:** 2024-07-09

**Authors:** Kaixun He, Haixiao Ding

**Affiliations:** College of Electrical Engineering and Automation, Shandong University of Science and Technology, Qingdao 266590, China; dinghx2023@163.com

**Keywords:** just-in-time learning, sample selection, random forest, thermal power plant, soft sensor, NOx

## Abstract

Combustion optimization is an effective way to improve the efficiency of thermal power generation and reduce carbon and NOx emissions. Real-time and precise NOx emission prediction is the basis for combustion optimization control of thermal power plants. To construct an accurate NOx concentration prediction model, a novel just-in-time learning (JITL) method based on random forest (RF) is proposed in the present work. With this method, first, an improved permutation importance algorithm is proposed to extract important variables. In addition, a similarity index that incorporates temporal and spatial measures is defined to select a local training set representative of the process data. Moreover, considering the influence of model parameters on prediction performance under different working conditions, a process monitoring method based on a moving window (MW) is used to monitor the change in working conditions and guide online updating. The experimental results show that the proposed method has excellent prediction accuracy, with a coefficient of determination of 0.9319, a root-mean-square error of 3.6960 mg/m^3^, and an average absolute error of 2.7718 mg/m^3^ on the test set, making it superior to other traditional methods.

## 1. Introduction

With the increasing attention paid to environmental protection, determining how to reduce carbon and toxic gas emissions in flue gas while improving boiler efficiency is an important and urgent problem to address in thermal power plants [1,2,3,4]. Over recent decades, the reliability of power grid supply has predominantly relied on thermal power generation, with coal-fired thermal power plants contributing approximately 60% of national electricity in China [5]. Furthermore, the integration of intermittent renewable energy sources like wind power into the grid hinges upon the peak-regulating capabilities of thermal power units [6]. Consequently, thermal power generation is poised to maintain its pivotal role over the long term. Coal combustion produces substantial quantities of toxic gases, notably NOx, a primary contributor to atmospheric pollution [7,8]. As stringent environmental regulations continue to be enacted, coal-fired power plants face progressively stringent NOx emission standards for flue gases. Currently, China’s laws stipulate that NOx emissions in flue gas should not exceed 50 $\mathrm{mg}/m^{3}$ [9], which poses a great challenge to the optimal control of power plant boilers and flue gas systems.

Accurate real-time detection of NOx concentration directly affects the amount of ammonia injected into the denitrification device, which is crucial for improving the efficiency of the selective catalytic reduction (SCR) and is the basis for boiler combustion optimization control [10]. The main chemical reaction equations of the denitration process are shown in Equations (1) and (2) [11].

(1) $$NO+NO_{2}+2NH_{3}\to 2N_{2}+3H_{2}O$$

(2) $$4NO+4NH_{3}+O_{2}\to 4N_{2}+6H_{2}O$$

As illustrated, insufficient ammonia injection can cause ammonia or NOx to escape, whereas excessive injection results in resource wastage. To deal with such problems, CEMS is widely used to track and monitor NOx concentration. Unfortunately, CEMS is greatly affected by environmental disturbances, resulting in inadequate detection accuracy and considerable lag [12]. In view of this challenge, the construction of a high-precision real-time NOx prediction model is a necessary supplement to existing detection methods [13]. Prediction strategies for NOx concentration mainly include model-based and data-based methods. The first method has better interpretation ability but requires a large amount of expert knowledge. The combustion process of thermal power plant boilers is highly complicated, making it difficult to establish accurate models for describing the process [14]. With the rapid development and application of distributed control systems (DCSs) in recent years, large amounts of industrial process data can be collected. As a result, data-driven methods have gradually emerged as a viable alternative. Compared with model-based methods, data-driven strategies bypass the need to solve complex conservation equations, resulting in faster and more reliable model responses; hence, they have received widespread attention. For example, Wang et al. [15] employed deep belief networks to extract data features and constructed networks consisting of an extreme learning machine (ELM), a backpropagation neural network (BPNN), and a radial basis function to predict NOx concentration. Jacob et al. [16] developed a combustion optimization system based on neural networks and particle swarm optimization, which effectively reduced the emission of NOx from power plants. Yang et al. [17] established a NOx prediction model based on a long short-term memory (LSTM) neural network, which exhibited better performance than least-squares support vector machine (LSSVM) and recurrent neural network models. Yuan et al. [18] proposed a NOx emission prediction method using linear regression as the metamodel and adopted BPNN, support vector regression, and decision tree as the basic models. The proposed stacked-generalization ensemble method demonstrated strong robustness and generalization capability. Li et al. [19] proposed a novel model architecture composed of a convolutional neural network and an effective channel attention module, which demonstrated good performance in predicting NOx emissions. Timo Korpela et al. [20] compared the performance of three nonlinear methods—multilayer perceptron, support vector regression, and fuzzy inference system—in predicting NOx concentration in natural gas boilers. Xie et al. [21] applied the sequence-to-sequence structure from the field of natural language processing to the LSTM model, achieving simultaneous prediction of NOx emissions at multiple time points. In addition, to reduce the impact of redundant variables on the performance of the prediction model, many variable selection methods have been incorporated. Xing et al. [22] utilized partial least squares (PLS) for variable selection and established an extreme gradient boosting ensemble model to predict the NOx emission concentration of coal-fired boilers. Tang et al. [23] employed mutual information combined with autoencoders and an ELM to deeply explore the relationship between NOx emission concentration and features. Wang et al. [24] utilized random forest (RF) to calculate the importance of variables and select the important process variables. Based on neural networks, Zhang et al. [25] employed the mean impact value to select variables and achieved multiobjective prediction of boiler thermal efficiency and NOx emission coupling. Tang et al. [12] utilized the LASSO and relief feature selection algorithms to select the important variables. They proposed an error correction strategy and established an ELM model, which can accurately predict NOx concentration at the boiler outlet. LSSVM [26,27], convolutional neural networks [28,29,30,31,32], and ensemble learning methods [33,34,35,36] have also been widely used by scholars in NOx concentration prediction.

Although the above studies achieved enhanced results under certain scenarios, they hardly meet the need for timely updates in online applications [3,37]. To address this issue, Lv et al. [38] improved LSSVM and updated the model based on an incremental strategy. Li et al. [14] proposed a variable exponentially weighted MWPLS method, which can be adaptively updated by adjusting the window size. Lara F. A. [39] used the $T^{2}$ and *Q* statistics of principal component analysis (PCA) to determine whether a model needs to be recalibrated. Lv et al. [40] proposed an adaptive strategy by updating the operating dataset when the model’s performance deteriorates. The above methods have achieved good results in addressing specific problems. However, the model parameters are difficult to adjust. Consequently, they are difficult to directly apply to the real-time prediction of NOx concentration in thermal power plants. Recently, just-in-time learning (JITL) [41,42] has seen widespread adoption due to its intrinsic characteristics favoring online implementation. Instead of building a global model, JITL creates a local model based on the similarity between input and output samples in real time. By using the current measured input data, similar samples in the database are collected for modeling. Considering the advantages of JITL, an improved scheme based on JITL for online prediction of NOx emissions is proposed in this work. With the proposed strategy, a supervised similarity distance measurement method is defined to adaptively select important training samples from the original dataset. RF regression is adopted to establish a prediction model, and an active updating strategy is proposed to maintain the model online. Moreover, to establish a robust model, a variable selection method is proposed that enables the robust selection of important variables. The industrial application results show that the presented strategy can provide good prediction accuracy and is suitable for long-term industrial applications.

This paper is organized as follows. Preliminaries about JITL and RF are provided in Section 2. Section 3 describes the target boiler in this work. Section 4 presents the proposed modeling method. Section 5 discusses the experiments and results obtained using real-world data. Finally, the conclusions are provided in Section 6.

## 2. Preliminaries

The key to predicting NOx concentration is to establish a learner f(·) with historical data. Subsequently, the auxiliary variable $X_{q}$ is substituted into the formula $\overset{⌢}{\;y}=f\left(X_{q}\right)+\varepsilon_{q}$ to obtain the prediction value. In this work, JITL and RF are combined to develop a local prediction model. To elucidate the proposed approach, this section outlines the foundational principles of JITL and RF.

### 2.1. JITL Method

JITL is a dynamic modeling framework in which all historical data are stored in a database, and a model is built in real time by searching the database for the samples most relevant to the query sample $X_{q}$ by a certain similarity index. After prediction, the established model is discarded. For the regression problem, the most common metric used to measure the similarity between $X_{q}$ and the historical sample $X_{i}$ is the Mahalanobis distance, which is defined as follows [43]:

(3) $$d_{M}\left(X_{i},X_{q}\right)=\sqrt{\left(X_{i}-X_{q}\right)\sum {\;}^{-1}{\left(X_{i}-X_{q}\right)}^{-1}}$$

where $X\in R^{n\times m}=\left\{X_{1},\cdots ,X_{i},\cdots X_{n}\right\}$, i=1,2,⋯,n denotes the sample number in the historical database and ∑ is the covariance matrix of *X*. Based on the Mahalanobis distance values, the similarity between $X_{q}$ and each sample in the historical data can be calculated using the Gaussian kernel function, as follows:

(4) $$S_{i,q}=\exp\left(-\frac{d_{M}^{2}\left(X_{i},X_{q}\right)}{2\sigma^{2}}\right)$$

where σ is the kernel width. In accordance with $S_{i,q}$, the first *l* samples with high similarity are selected from the historical data to construct the local training set. Then, the output prediction of the query sample $X_{q}$ is given by:

(5) $${\overset{⌢}{\;y}}_{q}\;=\;f(X_{q},\Theta )$$

where Θ is the hyperparameter of the model f(·).

### 2.2. RF Regression

**Figure 1:**
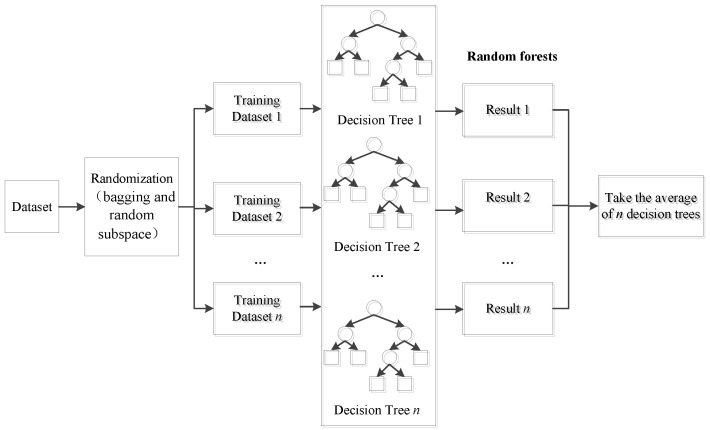
Schematic of RF regression modeling.

RF is an ensemble learning algorithm capable of generating numerous decision trees that serve as regression learners. The final prediction result of RF is derived from the mean value of all trees. The structure of RF is shown in Figure 1. Classification and regression tree (CART) is commonly employed as a decision tree for RF. Given a single output dataset $D=\{X_{train},Y_{train}\}$, where $X_{train}=\left(X_{1},X_{2},\cdots ,X_{N}\right)\in R^{N\times M}$, $Y_{train}=\left(y_{1},y_{2},\cdots ,y_{N}\right)\in R^{N\times 1}$, and *N* is the number of samples, along with the decision tree algorithm Γ and the number of base learners *T*, the steps for constructing a regression model using RF are as follows:

Step 1: For each decision tree *t*, *n* samples are randomly collected from *D* to construct a subtraining set $D_{t}$ using the bagging method, where t∈[1,T].

Step 2: The *t*th learner $h_{t}=\Gamma \left(D_{t}\right)$ is trained using $D_{t}$. During the training process, for each node, *m*(m<M) features are selected. Then, the optimal partition is selected from these *m* features to divide the molecular tree.

Step 3: During the formation of the decision tree, each node should be split in accordance with Step 2. The decision tree is trained with this subset until it is no longer possible to split, and the tree is not pruned.

Step 4: In accordance with Steps 1–3, a series of decision trees are established until *T* trees are trained.

During the application stage, the test samples are sent to each decision tree for regression prediction, and the prediction results of all decision trees for the same sample are counted. The average value of all results is used as the final predicted value, which can be expressed as follows:

(6) $$H=\frac{1}{T}\sum_{t=1}^{T}h_{t}(X)$$

The RF algorithm is well suited for modeling complex processes due to its effective utilization of randomness, which reduces inter-tree correlations. Therefore, in this work, RF regression is adopted as the basic modeling method.

## 3. Proposed Method

Generally, the representativeness of auxiliary variables determines the upper limit of model performance. Selecting important variables helps prevent dimensionality disasters and mitigate overfitting. With RF, important variables are typically identified using permutation importance. The basic idea of permutation importance is as follows: for each variable $x^{j}$, j∈[1,M]. The sequence of variables $x^{j}$ in out-of-bag (OOB) data is disrupted, and the corresponding relationship between auxiliary and output variables is broken. Subsequently, CART performs regression predictions on OOB data both before and after shuffling, calculating the mean square errors (MSEs) for each decision tree, which collectively determine the variable importance index $x^{j}$.

Given an RF with *T* decision trees $H=\{h_{1},h_{2},\cdots ,h_{t},\cdots ,h_{T}\}$, where t∈[1,T], the variable importance of $x^{j}$ is defined as follows:

(7) $$MSE=\frac{1}{n}\sum_{i=1}^{n}{(y_{i}-{\overset{⌢}{\;y}}_{i})}^{2}$$

(8) $${\bar{D}}_{j}=\frac{1}{T}\sum_{t=1}^{T}\left(MSE_{t}^{OOB}-MSE_{t,v}^{OOB}\right)$$

where $MSE_{t}^{OOB}$ and $MSE_{t,v}^{OOB}$ are the MSEs of the OOB data on tree *t* before and after interference, respectively, and ${\bar{D}}_{j}$ is the variable importance of $x^{j}$. The permutation importance algorithm of RF is presented in Algorithm 1.

**Algorithm 1**: The permutation importance algorithm of RF

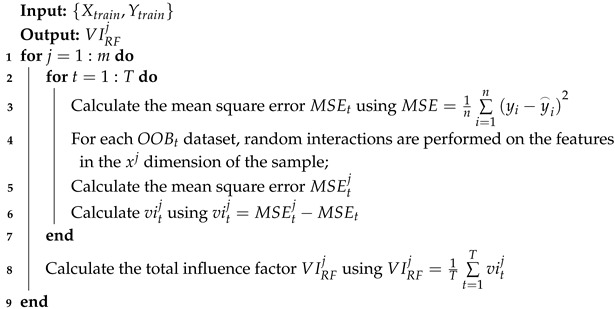



Based on the above permutation importance algorithm, determining the number of crucial variables to retain is not straightforward. In practice, threshold values are frequently empirically determined to facilitate variable selection, rendering the process notably subjective. To deal with this problem, this work proposes an iterative selection strategy, which determines the variable set to be retained by counting the frequency of selected variables in multiple runs. The specific steps of the improved variable selection method based on permutation importance are as follows:

Step 1: A regression model is established using each decision tree $h_{t}$, the value of the corresponding OOB data is predicted, and $MSE_{t}$ is output.

Step 2: For each $OOB_{t}$ set, the order of variable $x^{j},j\in \left[1,M\right]$, is shuffled to obtain a new set; then, $MSE_{t}^{j}$ is calculated using the model established in Step 1.

Step 3: The prediction influence factor of $x^{j}$ on tree $h_{t}$ is calculated using the following equation:

(9) $$vi_{t}^{j}=MSE_{t}^{j}-MSE_{t}$$

Step 4: All decision trees are traversed, and Steps 1–3 are repeated. Afterward, the influence factors of $x^{j}$ on all decision trees are obtained.

Step 5: The total influence factor of $x^{j}$ on the RF is calculated as follows:

(10) $$VI_{RF}^{j}=\frac{1}{T}\sum_{t=1}^{T}vi_{t}^{j}$$

Step 6: All variables in $X_{train}$ are traversed, and Steps 1–5 are repeated to acquire the influence factors $VI_{RF}$ of all variables.

Step 7: The variables are sorted in accordance with the value of $VI_{RF}^{j}$.

Step 8: Steps 1–7 are repeated *ℓ* times, and the variables with the highest frequency in the top *K* are saved.

Step 9: The variable with the lowest frequency of 10% is eliminated each time, and the corresponding OOB error after removing the variables is calculated.

Step 10: The subset of variables corresponding to the minimum OOB error is selected.

### 3.1. Strategy for Local Training Sample Selection

In JITL, a crucial initial task involves identifying local training samples that closely resemble the query sample. To implement this operation, the distance $d_{M_{i}}$ between the query sample $X_{q}$ and the *i*th historical sample $X_{i}$ is defined first, and then local training samples are selected based on $d_{M_{i}}$. As mentioned above, the Mahalanobis distance is generally used to define the similarity for regression modeling. The Mahalanobis distance considers the spatial characteristics of auxiliary variables but ignores the relationship between dependent variables.

Due to the strong time sequence characteristics inherent in thermal power plant process data, a more representative similarity index can be constructed by introducing the dependent variable information of the adjacent time-domain samples. Accordingly, we propose an improved similarity index by simultaneously considering the temporal and spatial characteristics of the independent and dependent variables.

Using the proposed scheme, the Mahalanobis distance $d_{M_{i}}$ between $X_{i}$ and $X_{q}$ is calculated. The similarity index of all samples in the historical dataset is obtained. The average value of the NOx concentration of the sample in the preceding Mw time window of $X_{q}$ is calculated. The distance of the dependent variable is calculated as follows:

(11) $$d_{y}\left(y_{i},{\bar{y}}_{local}\right)=\sqrt{{\left(y_{i}-{\bar{y}}_{local}\right)}^{2}}$$

where $y_{i}$ is the concentration of NOx in the historical data and ${\bar{y}}_{local}$ is the average concentration within the Mw time scale.

In this way, the distance $d_{M}$ defined by the independent variable and the distance $d_{y}$ defined by the temporal data and dependent variables are obtained. The final similarity can be calculated through the following equations:

(12) $$d_{sim}\left(X_{i},X_{q}\right)=\lambda d_{M}+\left(1-\lambda \right)d_{y}$$

(13) $$S_{i,q}=\exp\left(-\frac{d_{sim}^{2}\left(X_{i},X_{q}\right)}{2\sigma^{2}}\right)$$

where 0≤λ≤1 is a conversion factor; when λ=1, the similarity degenerates to a method based solely on the Mahalanobis distance.

In accordance with the above method, *L* samples with high similarity can be selected from the historical data to construct a local training set $D_{l}$. Given that the local training set selected using the above method still has high leverage points, the existence of such samples reduces the performance of the constructed prediction model. At the end of sample selection, we use an *F* distribution to eliminate sample points with low confidence. The specific operations are described below.

The mean value of the independent variables in $D_{l}$ is calculated using Equation (14). If $d_{M}\left(X_{l},u\right)>F_{\alpha }\left(M,L\right)$, then the sample $\left\{X_{l},Y_{l}\right\}$ is deleted from $D_{l}$, where α is the quantile; *M* and *L* are the number of independent variables and the number of samples in $D_{l}$, respectively. For ease of description, the final selected local sample set is still represented by $D_{l}$.

(14) $$u=mean(X_{local})$$

The pseudocode for local training sample selection based on the proposed similarity definition method is shown in Algorithm 2.

**Algorithm 2**: The algorithm for local training sample selection

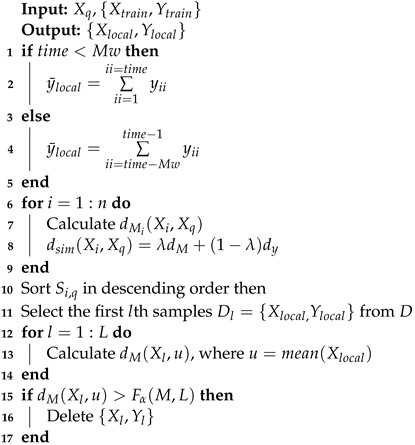



### 3.2. Process Condition Monitoring and Parameter Updating Strategy

The thermal power generation process has typical multicondition characteristics. Under the same working conditions, a high-performance prediction model can be constructed using JITL. However, the local training set constructed according to Equation (12) may drift in its representation of the process conditions after switching conditions. To address this problem, MWPCA is adopted to monitor the production process. Then, λ is updated timely in accordance with the monitoring results. The specific process of the algorithm is described below.

The moving window length is denoted as *L*, and the corresponding data matrix within the window at time *t* is denoted as MatrixI: $X_{t}=(x_{t-L+1},x_{t-L+2},\cdots ,$ $x_{t-1},x_{t}{)}^{T}\in R^{L\times M}$. At time t + 1, the corresponding data matrix within the window is denoted as MatrixIII: $X_{t+1}={\left(x_{t-L+2},x_{t-L+3},\cdots ,x_{t},x_{t+1}\right)}^{T}\in R^{L\times M}$, and the data matrix corresponding to the transition window is denoted as MatrixII: ${\tilde{X}}_{t,t+1}={\left(x_{t-L+2},x_{t-L+3},\cdots ,x_{t-1},x_{t}\right)}^{T}\in R^{(L-1)\times M}$. The specific steps are as follows:

Step 1: Transition from MatrixI to MatrixII:

$\mu_{t}\in R^{M\times 1}$ and $C_{t}\in R^{M\times M}$ are set as the mean vector and covariance matrix of MatrixI, respectively. The mean vector ${\tilde{\mu }}_{t,t+1}$ and standard deviation $\delta_{t,t+1}^{i}\left(i=1,2,\cdots ,\;M\right)$ of the variables for MatrixII can be determined using Equations (15) and (16), respectively.

(15) $${\tilde{\mu }}_{t,t+1}=\frac{L}{L-1}\mu_{t}-\frac{1}{L-1}x_{t-L+1}$$

(16) $${\left(\delta_{t,t+1}^{i}\right)}^{2}=\frac{L-1}{L-2}{\left(\delta_{t}^{i}\right)}^{2}-\frac{L-1}{L-2}{\left(\mu_{t}^{i}-{\tilde{\mu }}_{t,t+1}^{i}\right)}^{2}-\frac{1}{L-2}\|{x^{i}}_{t-L+1}-\mu_{t}^{i}{\|}^{2}$$

(17) $$\sum_{t,t+1}^{\;}=diag\left(\delta_{t,t+1}^{1},\delta_{t,t+1}^{2},\cdots ,\delta_{t,t+1}^{M}\right)$$

The covariance matrix ${\tilde{C}}_{t,t+1}\in R^{M\times M}$ of MatrixII can be derived from the above formula and is shown below:

(18) $${\tilde{C}}_{t,t+1}=\frac{L-1}{L-2}\left(C_{t}-\frac{1}{L-1}{x^{T}}_{t-L+1}x_{t-L+1}-\sum_{t}^{-1}\left(\mu_{t}-{\tilde{\mu }}_{t,t+1}\right){\left(\mu_{t}-{\tilde{\mu }}_{t,t+1}\right)}^{T}\sum_{t}^{-1}\right)$$

where $\sum_{t}^{\;}=diag\left(\delta_{t}^{1},\delta_{t}^{2},\cdots ,\delta_{t}^{M}\right)$ is the diagonal matrix composed of the standard deviations of each variable in MatrixI.

Step 2: Transition from MatrixII to MatrixIII:

Similar to Step 1, here, the mean vector $\mu_{t+1}$ of MatrixIII and the standard deviation $\delta_{t+1}^{i}\left(i=1,2,\cdots ,M\right)$ of each variable can be recursively calculated.

(19) $$\mu_{t+1}=\frac{L-1}{L}{\tilde{\mu }}_{t,t+1}+\frac{1}{L}x_{k+1}$$

(20) $${\left(\delta_{t+1}^{i}\right)}^{2}=\frac{L-2}{L-1}{\left(\delta_{t,t+1}^{i}\right)}^{2}+{\left(\mu_{t+1}^{i}-{\tilde{\mu }}_{t,t+1}^{i}\right)}^{2}-\frac{1}{L-1}\|{x^{i}}_{t+1}-\mu_{t+1}^{i}{\|}^{2}$$

(21) $$\sum_{t+1}^{\;}=diag\left(\delta_{t+1}^{1},\delta_{t+1}^{2},\cdots ,\delta_{t+1}^{M}\right)$$

The new samples are standardized as follows:

(22) $$x_{t+1}=\sum_{t+1}^{-1}(x_{t+1}-\mu_{t+1})$$

The covariance matrix $C_{t+1}\in R^{M\times M}$ of MatrixIII can be derived from the above formula.

(23) $$C_{t+1}=\frac{L-2}{L-1}{\tilde{C}}_{t,t+1}+\frac{1}{L-1}{x^{T}}_{t+1}x_{t+1}+\sum_{t+1}^{-1}\left(\mu_{t+1}-{\tilde{\mu }}_{t,t+1}\right){\left(\mu_{t+1}-{\tilde{\mu }}_{t,t+1}\right)}^{T}\sum_{t+1}^{-1}$$

Substituting Equation (18) into Equation (23) yields the expression for transitioning from MatrixI to MatrixIII as follows:

(24) $$\begin{array}{c} C_{t+1}=C_{t}-\sum_{t}^{-1}(\mu_{t}-{\tilde{\mu }}_{t,t+1}){\left(\mu_{t}-{\tilde{\mu }}_{t,t+1}\right)}^{T}\sum_{t}^{-1}\\ -\frac{1}{L-1}x_{t-L+1}{x^{T}}_{t-L+1}+\frac{1}{L-1}x_{t+1}x_{t+1}^{T}\\ +\sum_{t+1}^{-1}(\mu_{t+1}-{\tilde{\mu }}_{t,t+1}){\left(\mu_{t+1}-{\tilde{\mu }}_{t,t+1}\right)}^{T}\sum_{t+1}^{-1} \end{array}$$

After the covariance matrix of the data in the new window is obtained, the corresponding principal component model can be obtained through singular value decomposition of the covariance matrix $C_{t+1}$. The statistic $T^{2}$ and the control limit for $T^{2}$ with confidence level α can be determined using Equations (25) and (26), respectively.

(25) $$T^{2}=x^{T}P\Lambda^{-1}P^{T}x$$

(26) $$T_{\alpha }^{2}=\frac{A(L^{2}-1)}{L(L-A)}F_{A,L-a;\alpha }$$

where $\Lambda \in R^{A\times A}$ is the diagonal matrix composed of the first *A* eigenvalues of the covariance matrix $C_{t+1}$ and $F_{A,L-a;\alpha }$ is the critical value of the *F* distribution with *A* and L−A degrees of freedom and a significance level of α.

When the $T^{2}$ corresponding to the query sample is greater than the monitoring threshold, a working condition switch has occurred. If the monitoring statistic of the query sample does not exceed the monitoring threshold, the historical data in the moving window have characterized the current process well. In this case, λ can take a slightly smaller value, and vice versa.

### 3.3. Overall Flow of the Proposed Modeling Strategy

**Figure 2:**
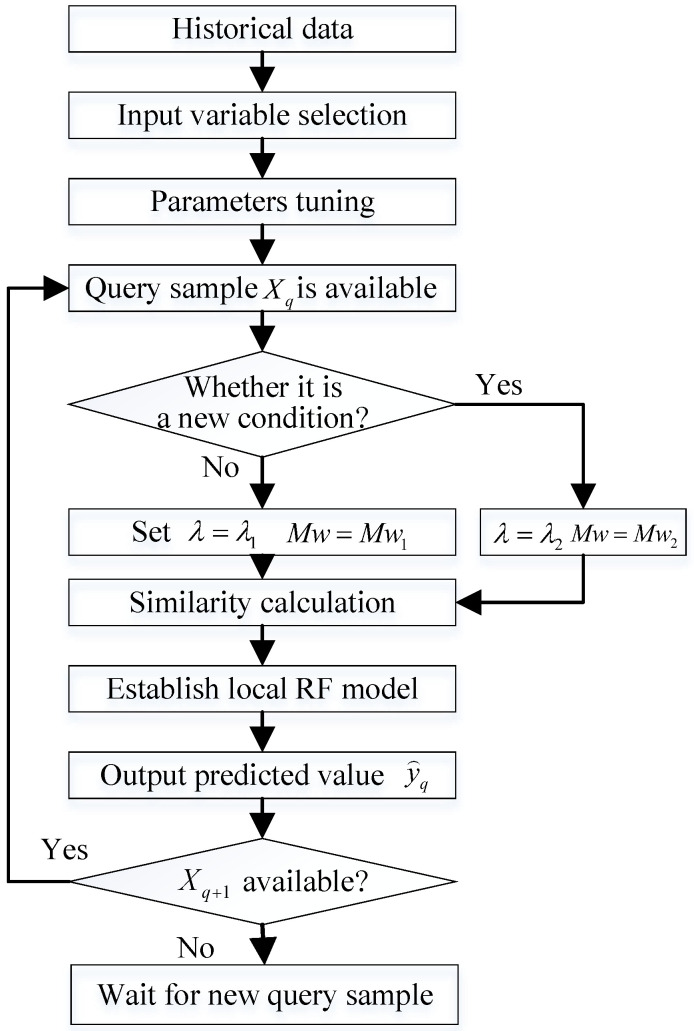
Basic concept of the proposed JIT-RF modeling framework.

Following the aforementioned variable selection and local training sample selection methods, the overall flow of the NOx prediction model based on JITL is presented in Figure 2, and the corresponding pseudocode is shown in Algorithm 3.

**Algorithm 3**: Just-in-time RF

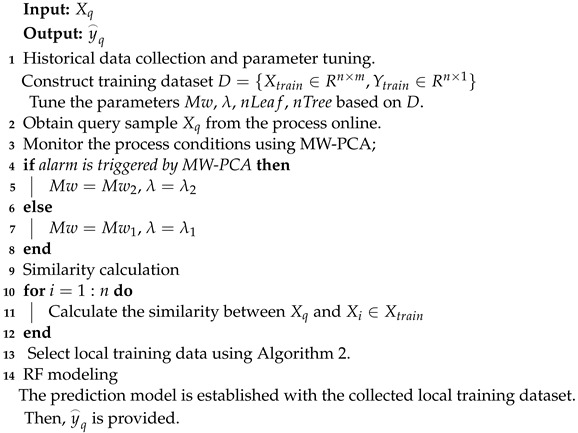



## 4. Boiler System and Data Preparation

### 4.1. Description of the Boiler System

**Figure 3:**
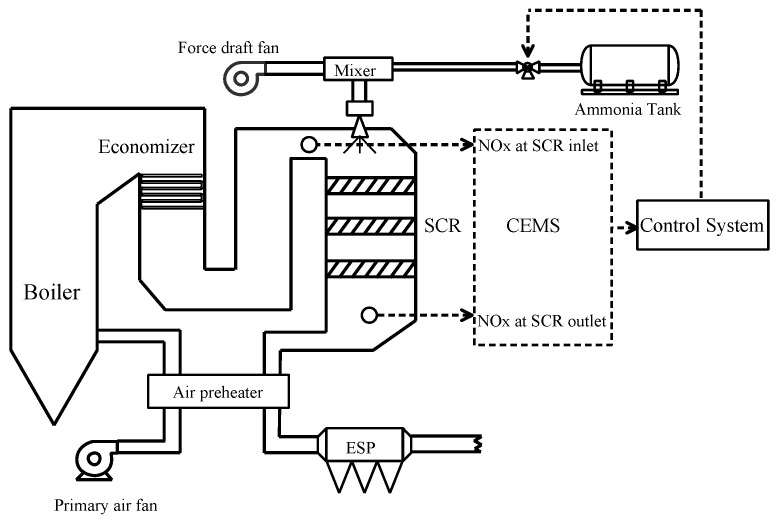
Process flow of SCR denitrification.

In this work, the direct current boiler of a 1030 MW ultra-supercritical coal-fired unit is adopted as the target object. The object is a π-type boiler, featuring balanced ventilation, ultra-supercritical parameters, primary reheat, a spiral furnace, solid slag discharge, and an open-air arrangement. The pulverizing system employs a medium-speed coal mill with positive-pressure direct cooling primary air. The combustion unit adopts a front and rear wall hedge combustion mode, featuring low-NOx double adjustable air swirl burners and nozzles. The furnace dimensions are 64,500 mm in height, 33,128.7 mm in cross-sectional width, and 16,308.7 mm in depth. A flue gas-regulating baffle device is arranged at the bottom of the flue passage to distribute the flue gas, maintaining the reheat steam outlet temperature within the control load range. The flue gas is collected by the regulating baffle and then introduced into the SCR denitration device through the two tail flues. After denitration, the flue gas enters the air preheater. Figure 3 shows the overall structure of the boiler and SCR denitrification.

### 4.2. Data Description

The historical data used in this work were obtained from DCS, and a total of 5184 groups of 72 h data were collected at a sampling interval of 50 s. The abnormal samples were eliminated using the 3σ rule (Equation (27)), and 5174 groups of data were retained.

(27) $$\bar{y}-3\sigma \leq y\leq \bar{y}+3\sigma$$

where σ and $\bar{y}$ represent the standard deviation and mean value of NOx concentration, respectively. Among these data, 30% were randomly selected to construct the training set, about 60% were used as the test set, and the remaining 10% were utilized to construct the verification set. The original data contained 390 auxiliary variables, which were normalized as follows:

(28) $$x=\frac{x-\bar{x}}{\varphi }$$

where $\bar{x}$ and φ represent the mean and standard deviation values, respectively.

To assess the performance of the mentioned models, three indices—root-mean-square error (RMSE), coefficient of determination (R^2^), and mean absolute error (MAE)—were adopted. Generally, the smaller the values of the RMSE and MAE, the higher the accuracy of the model. R^2^ describes the explanatory ability; the closer its value to 1, the greater the explanatory ability of the model.

(29) $$RMSE=\sqrt{\frac{1}{n}\sum_{i=1}^{n}{(y_{i}-{\overset{⌢}{\;y}}_{i})}^{2}}$$

(30) $$R^{2}=1-\frac{\sum_{i=1}^{n}{(y_{i}-{\overset{⌢}{\;y}}_{i})}^{2}}{\sum_{i=1}^{n}{(y_{i}-{\overset{¯}{\;y}}_{i})}^{2}}$$

(31) $$MAE=\frac{1}{n}\sum_{i=1}^{n}\left|y_{i}-{\overset{⌢}{\;y}}_{i}\right.$$

where *n* is the number of test samples; $y_{i}$ and ${\overset{⌢}{\;y}}_{i}$ represent the actual and predicted values, respectively; and denotes the mean value.

## 5. Case Study

### 5.1. Parameter Tuning

**Figure 4:**
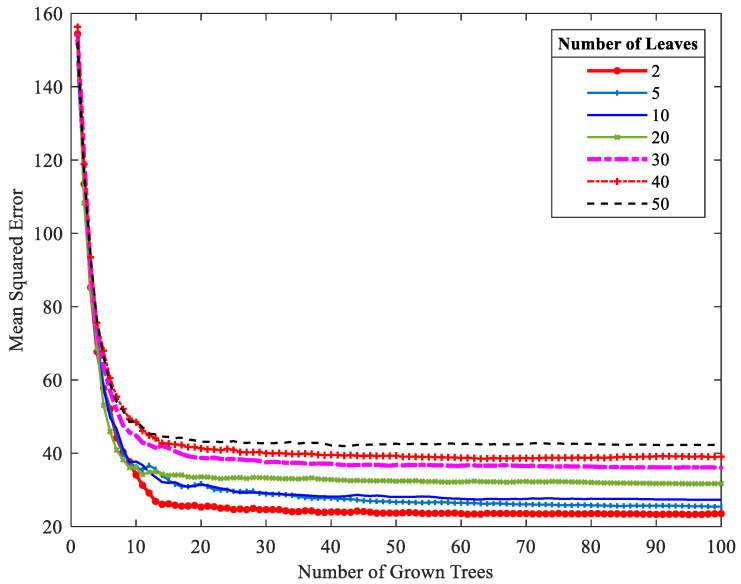
OOB error curves with varying values of nLeaf and nTree.

Two parameters should be adjusted when constructing a model using RF: the number of leaf nodes, i.e., nLeaf, and the number of decision trees, i.e., nTree. In this work, these parameters are adjusted based on the OOB error on the training set. The numbers of leaf nodes and decision trees that minimize the OOB error are regarded as the optimal parameters. As illustrated in Figure 4, when nLeaf is 2, the corresponding MSE curve is the lowest. After the number of decision trees is increased to more than 50, the MSE of the OOB samples hardly decreases. Therefore, in this work, the values of nLeaf and nTree are set to 2 and 50, respectively.

### 5.2. Results and Discussion

**Figure 5:**
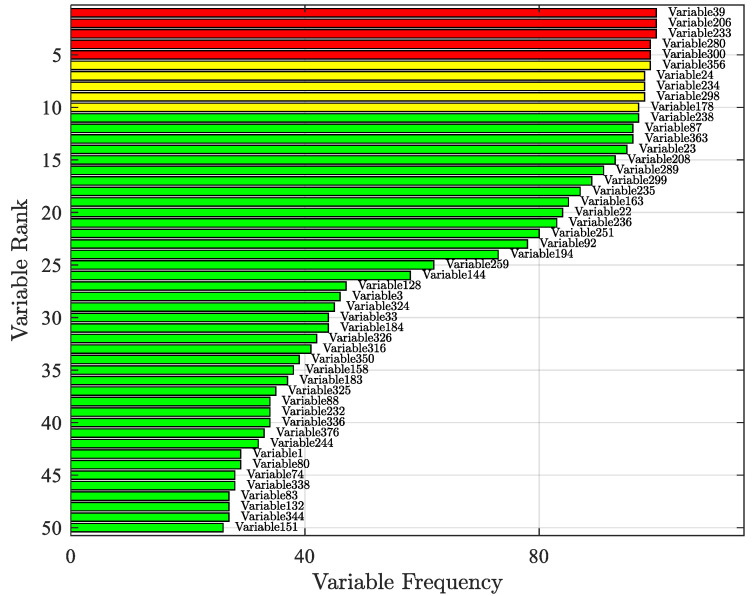
Variable selection results.

In accordance with the variable selection method mentioned above, with ℓ=100 and *K* = 50, the RF model was run 100 times independently. Subsequently, variables that most frequently entered the top 50 based on their importance index were tallied. Finally, a total of 38 sets of variables were selected. Figure 5 shows the selection results.

**Figure 6:**
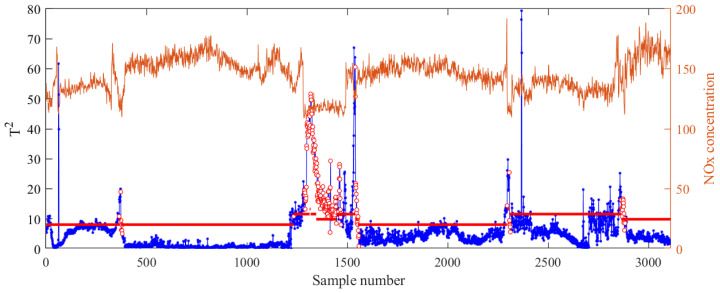
MW-PCA monitoring results.

To improve adaptability to the multicondition process, MW-PCA was employed to determine whether the parameter λ needed to be updated. Experimentally, the window size was set to Mw = 200 and monitored for 20 consecutive times. When the $T^{2}$ control limit was exceeded 15 times or more, an alarm signal was generated. This signal indicated that the process conditions had changed, and the parameter λ had to be updated. Figure 6 illustrates the monitoring results of MWPCA on the test set (the thick red line represents the $T^{2}$ control limit for each window, the blue line represents the calculated $T^{2}$ and the red circle represents the update time of the model).

As illustrated in Figure 6, a total of 228 changes were detected. The alarm occurred frequently— four times. These alarm messages were concentrated in periods of significant fluctuation in NOx concentration. The greater the fluctuation in NOx concentration, the more alarms occurred. These periods corresponded to transitional conditions before and after process changes. When the process condition was switched, ${\bar{y}}_{local}$ in Equation (11) was insufficient as a representative for the query sample $y_{q}$. Therefore, λ should take a slightly larger value to ensure that the similarity calculated using Equation (12) maintains good accuracy.

**Figure 7:**
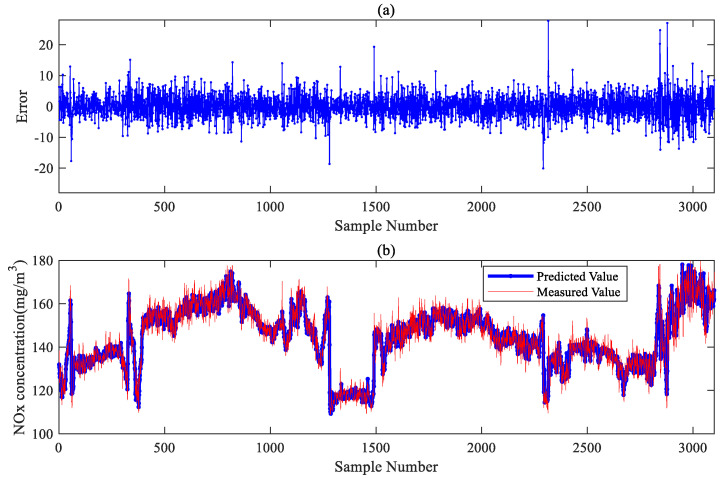
Prediction and error curves of the proposed method. (**a**) The prediction error of the proposed method on the test set. (**b**) The predicted results of the proposed method on test set.

Figure 7a presents the errors between the measured and predicted values of NOx, predominantly concentrated within the range of ±10 $\mathrm{mg}/m^{3}$. This indicates that the model demonstrates excellent stability and accuracy. Figure 7b intuitively shows the situation between the predicted and actual values. The model generally tracked NOx concentration variations well, albeit with large errors when the NOx concentration fluctuated significantly. These moments generally corresponded to transitional stages of changes in the operating conditions. This finding is consistent with Figure 6, where the model exhibited significant errors during changes in the operating conditions. Subsequent updates incorporating the latest operational data resulted in improved and sustained model accuracy.

**Figure 8:**
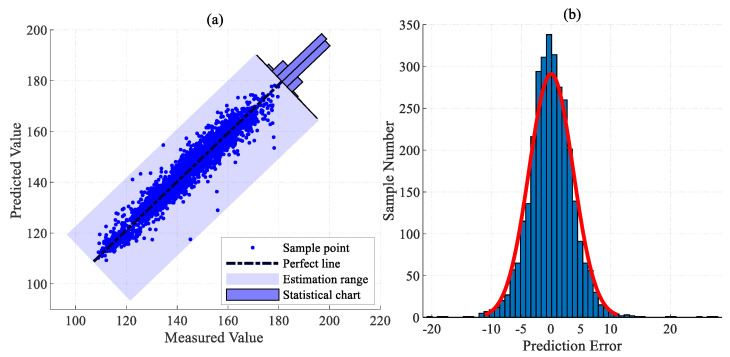
Scatter plot and error distribution diagram of the proposed model. (**a**) The scatter plot of actual measured and predicted values. (**b**) The distribution map of prediction error.

Figure 8a shows the scatter plot of the predictions. The predicted and measured values are closely distributed near the perfect straight line, with small deviation and variance, demonstrating the excellent predictive performance of the model. Figure 8b indicates that the model prediction errors are primarily concentrated within the range of —5 $\mathrm{mg}/m^{3}$ to 5 $\mathrm{mg}/m^{3}$, with 99.71% of samples exhibiting an absolute error within 15 $\mathrm{mg}/m^{3}$.

To validate the superiority of the proposed method, the traditional PLS and RF models, the MW-PLS and MW-RF models incorporating the MW strategy, and the JIT-PLS and JIT-RF models with the JITL strategy were adopted as comparison methods. Table 1 presents the results of the three evaluation metrics for the six models and the proportion of samples with an absolute error within 15 $\mathrm{mg}/m^{3}$.

The R^2^, RMSE, and MAE values of the proposed model on the test set were 0.9319, 3.6960 $\mathrm{mg}/m^{3}$, and 2.7718 $\mathrm{mg}/m^{3}$, respectively, surpassing those of the other models in both prediction accuracy and error distribution. The predictive performance of the RF model was superior to that of the PLS model, which indicates that the RF model holds a greater advantage in predicting NOx concentration. The predictive performance of the PLS and RF models with the addition of the MW strategy was inferior to that of the traditional PLS and RF models, possibly due to information loss caused by the fixed length of MW. Specifically, the R^2^, RMSE, and MAE of the PLS model on the test set were 0.7879, 6.5226 $\mathrm{mg}/m^{3}$, and 4.5792 $\mathrm{mg}/m^{3}$, respectively. The R^2^, RMSE, and MAE of the JIT-PLS model on the test set were 0.8473, 5.5345 $\mathrm{mg}/m^{3}$, and 3.7936 $\mathrm{mg}/m^{3}$, respectively. A comparison of the performance of the PLS and JIT-PLS models indicates that the PLS model incorporating the JITL strategy exhibited better predictive performance compared to the traditional PLS model, which proves the effectiveness and applicability of the JITL strategy. The performance results of the JIT-RF model on the test set were 0.9252, 3.8727 $\mathrm{mg}/m^{3}$, and 2.9018 $\mathrm{mg}/m^{3}$. Incorporating the model update strategy into the JIT-RF model leads to further improvements in prediction performance.

**Figure 9:**
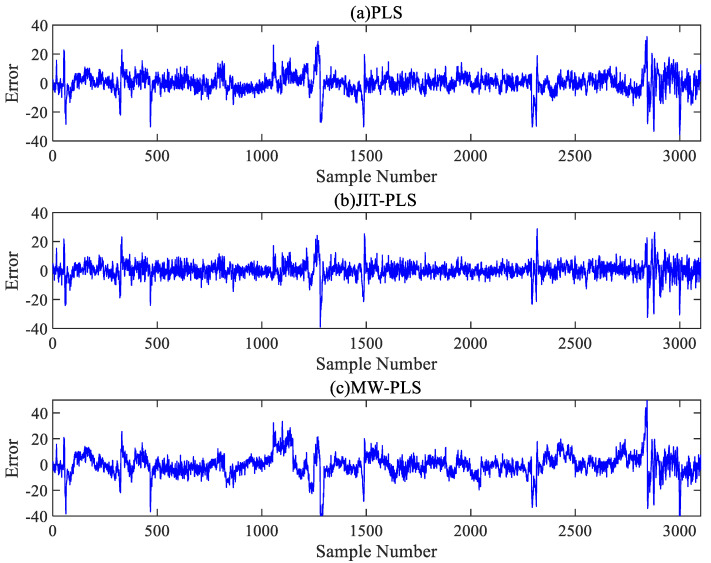
Error curves of the PLS algorithm.

**Figure 10:**
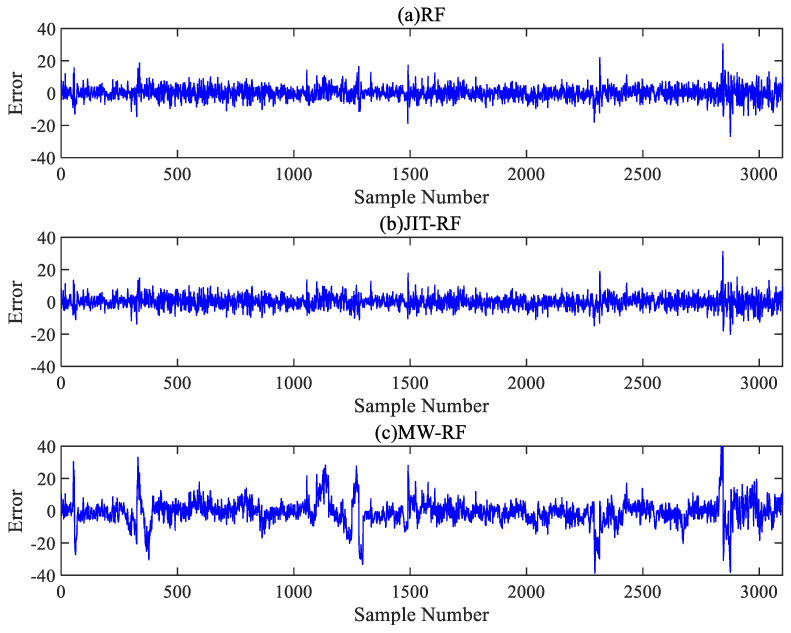
Error curves of the RF algorithm.

Figure 9 and Figure 10 show the error distributions of the six comparative algorithms on the test set, illustrating that the RF model exhibited more stable prediction performance compared to the PLS model, with the JIT-RF model demonstrating the highest prediction accuracy. The incorporation of the JITL strategy into both the PLS and RF models resulted in a significant reduction in prediction errors. The range of error fluctuation also significantly improved, indicating the effectiveness of the JITL strategy. Conversely, the inclusion of the MW strategy led to a notable increase in prediction errors and the fluctuation range of errors. Thus, the addition of the MW strategy results in decreased accuracy due to the loss of input information and increased instability in prediction.

**Figure 11:**
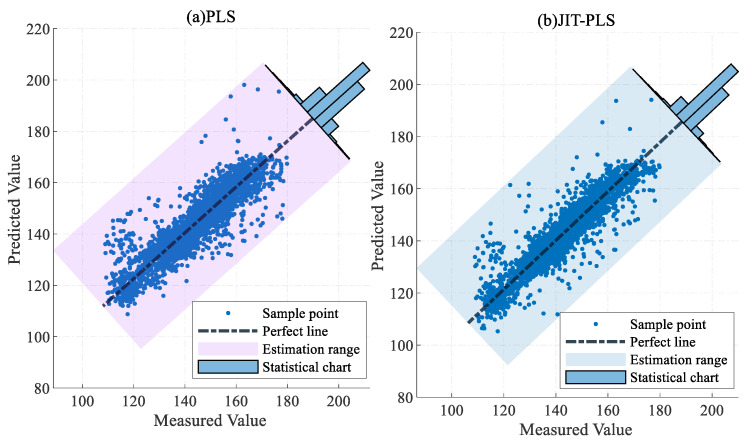
Predictive scatter plots for PLS and JIT-PLS models.

**Figure 12:**
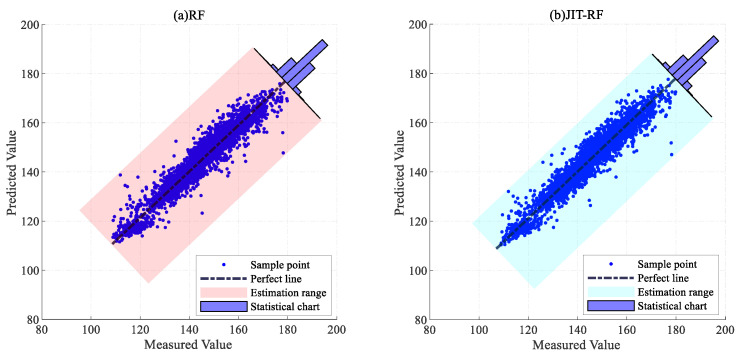
Predictive scatter plots for RF and JIT-RF models.

**Figure 13:**
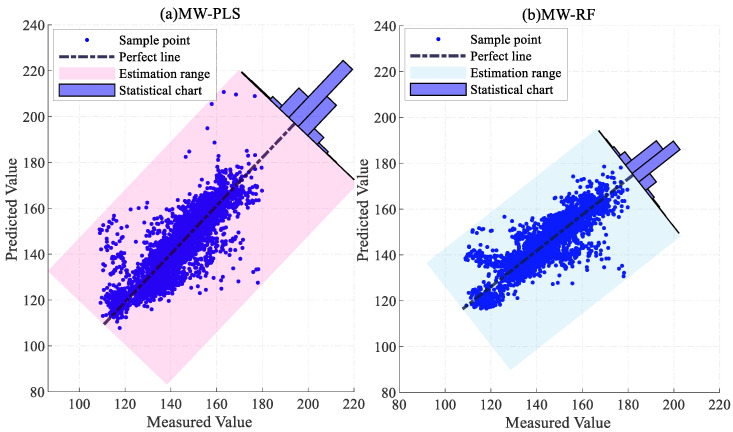
Predictive scatter plots for MW-PLS and MW-RF models.

Figure 11, Figure 12 and Figure 13 present the error statistics and scatter plots. In Figure 12b, the NOx concentration predicted by the JIT-RF model and the actual measured values approximately follow the diagonal line. In contrast, Figure 13a demonstrates that the sample points predicted by the MW-PLS model deviate significantly from the perfect line. MW-PLS exhibited the worst and most unstable predictive performance, whereas the JIT-RF model had better predictive accuracy than the other models. After the addition of the MW strategy, the predictive performance of the MW-PLS and MW-RF models decreased (Figure 13), possibly due to the incomplete information in the training set caused by the MW strategy. After the JITL strategy was incorporated into the PLS and RF models, the sample points became closer to the perfect line (Figure 11b and Figure 12b), indicating that the prediction performance of the model improved and demonstrating the advantages of the JITL strategy.

The above analysis indicates that the proposed method outperforms traditional approaches and is particularly suited for the online prediction of the concentration of NOx emissions.

## 6. Conclusions

In this work, an improved JITL-based prediction method is proposed to predict the concentration of NOx emissions in a coal-fired power plant. A supervised similarity distance measurement method is defined, and local training samples can be effectively selected. To establish a robust model, a variable selection method is also proposed that enables the robust selection of important variables. Several comparative experiments on a real-world industrial dataset are presented. The experiments show that the proposed method has good accuracy. Therefore, it is suitable for the long-term prediction of NOx emissions. With the development of industrial information technology, the acquisition of multisource process data has become increasingly convenient. Based on multisource data, such as audio and images, building a large prediction model will be beneficial to improving the accuracy and real-time performance of NOx emission prediction, which will be our future research focus.

## Figures and Tables

**Table 1:** Prediction results of different models.

Method	R^2^	RMSE	MAE	Proportion with Absolute Error Less than 15 $\mathrm{mg}/m^{3}$
JIT-PLS	0.8473	5.5345	3.7936	97.29%
PLS	0.7879	6.5226	4.5792	96.23%
MW-PLS	0.6222	8.7057	6.1037	92.48%
RF	0.9197	4.0138	2.9939	99.58%
MW-RF	0.7095	7.6345	5.1912	93.94%
JIT-RF	0.9252	3.8727	2.9018	99.68%
Proposed method	0.9319	3.6960	2.7718	99.71%

## Data Availability

The data generated in this study are presented in this article. For any clarifications, please contact the corresponding author.
